# Accidental transcutaneous displacement of a fiducial marker during transperineal prostate biopsy: a case report and narrative review

**DOI:** 10.1186/s12894-026-02125-1

**Published:** 2026-03-25

**Authors:** Charlotte Müssgens, Beat Roth, Nicolas Arnold, Laila Schneidewind, Mohamed Shelan, Nicola Giudici

**Affiliations:** 1 Department of Urology, Inselspital, Bern University Hospital, University of Bern, Bern, Switzerland; 2 Department of Radiation Oncology, Inselspital, Bern University Hospital, University of Bern, Bern, Switzerland

**Keywords:** Case report, Prostate cancer, Fiducial markers, Transperineal biopsy, Marker displacement, Radiotherapy planning

## Abstract

**Background:**

Fiducial markers are commonly used to guide precise radiotherapy in prostate cancer patients. While intraprostatic migration has been described, transcutaneous displacement during biopsy is exceedingly rare. We report the first known case of transcutaneous fiducial marker removal during transperineal prostate rebiopsy.

**Case Presentation:**

A 62-year-old man with high-risk prostate cancer underwent radiotherapy with gold fiducial markers in 2020. Following two biochemical recurrences, transperineal MRI/ultrasound fusion-guided biopsy was performed in 2024. Increased resistance was encountered during the final biopsy pass, and a gold marker was found embedded in the core sample and was inadvertently removed transcutaneously. The procedure was well tolerated, and no complications occurred.

**Conclusions:**

This case illustrates that even long after implantation, fiducial markers may still become dislodged during prostate interventions. While the event was asymptomatic, it highlights the importance of procedural awareness and planning to prevent potential compromise of radiotherapy accuracy in future cases.

## Background

Prostate cancer (PCa) is the most commonly diagnosed malignancy in men in Western countries, and radiotherapy—alongside surgery—represents one of the primary treatment options for patients with clinically significant disease [1].

Image-guided radiotherapy (IGRT) is a pivotal advancement in the precise delivery of modern radiotherapy techniques. While aligning prostate radiotherapy via skin marks or bony anatomy may be sufficient for administering a relatively uniform whole-pelvic dose, these methods fall short when reduced margins, dose escalation, or hypofractionated stereotactic radiotherapy are employed. To increase targeting accuracy, fiducial markers have been utilized for many decades [2]. This approach allows for reduced safety margins, thereby minimizing radiation exposure to surrounding healthy tissues and decreasing the risk of complications, while maintaining effective tumor control and facilitating dose escalation [3].

Gold fiducial marker placement is a routine urological procedure. Traditionally, these markers are implanted via a transrectal approach. However, over the past decade, the transperineal approach has gained prominence, particularly for prostate biopsies, owing to its lower risk of postprocedural infections and improved sampling of the anterior prostate zones [4]. Consequently, the transperineal route is increasingly considered a safe and well-tolerated alternative for fiducial marker placement, potentially offering advantages in infection prevention [5].

In this case report, we describe an instance of iatrogenic transcutaneous displacement and removal of a gold fiducial marker during transperineal prostate rebiopsy.

## Case presentation

A 62-year-old male with a known history of high-risk prostate adenocarcinoma was referred to our multidisciplinary tumor board due to suspected PCa local recurrence.

The initial diagnosis in 2020 revealed prostate adenocarcinoma stage cT2c cN0 cM0, with a Gleason score of 4 + 5=9. After transrectal placement of two fiducial markers, the patient underwent external beam radiotherapy at a total dose of 74 Gy, combined with androgen deprivation therapy (ADT) for six months.

The post-treatment PSA nadir was < 0.03 ng/ml. Biochemical recurrence was first documented in July 2023 with a PSA increase to 2.1 ng/ml. PSMA-PET/CT demonstrated findings consistent with local recurrence. Multiparametric MRI revealed two suspicious lesions (PI-RADS 5) located in the anterior mid-gland transitional zone (right) and the posterior mid-gland peripheral zone (left). Rebiopsy confirmed recurrent disease bilaterally, with a Gleason score of 5 + 5 = 10.

Salvage stereotactic body radiotherapy was administered in November 2023, and was delivered in five fractions of 6 Gy (total 30 Gy) via CyberKnife^®^ using the pre-existing fiducial markers for treatment tracking. Concomitant ADT was continued for six months with good tolerability.

At the three-month follow-up, the PSA level had declined to 0.08 ng/ml. The patient reported satisfactory urinary function.

**Fig. 1:**
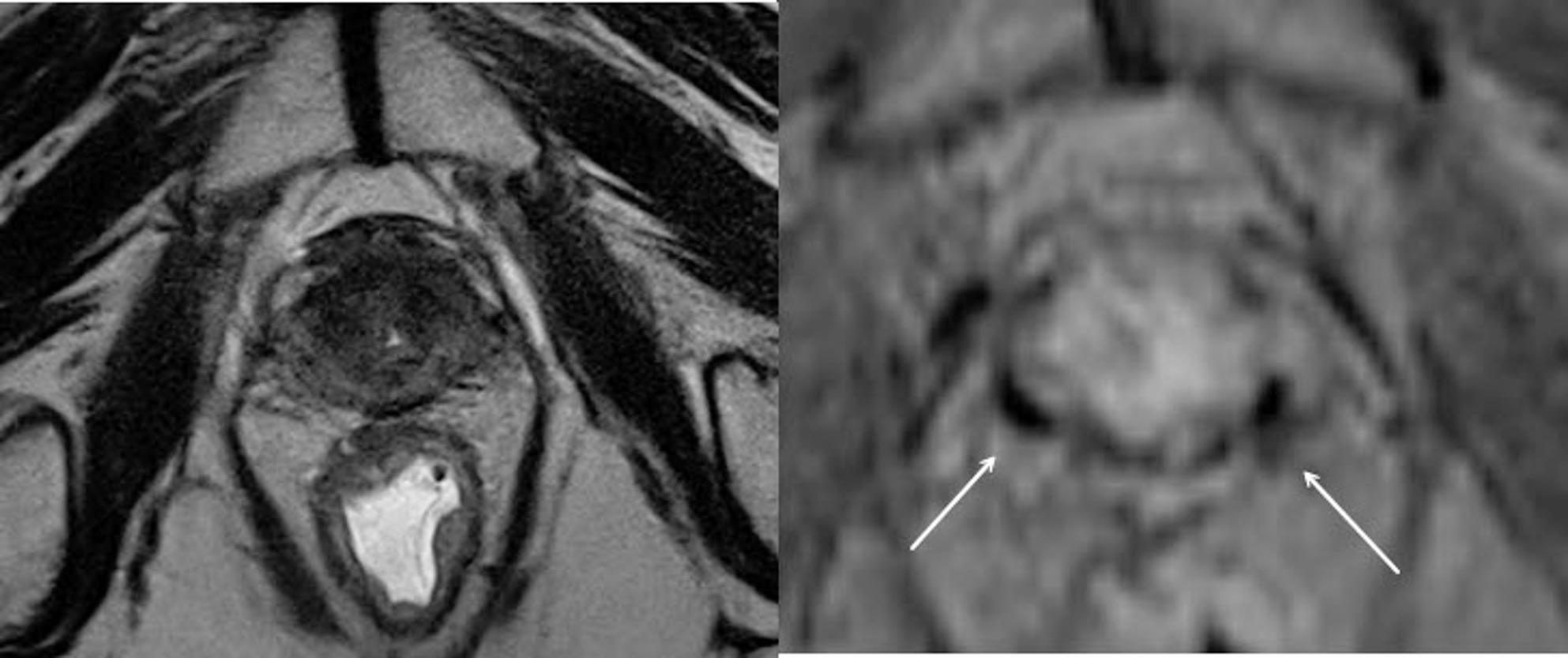
Prostate MRI before biopsy. Left: T2-weighted sequence; right: T1-weighted sequence. The two white arrows indicate the two hypointense gold markers

**Fig. 2:**
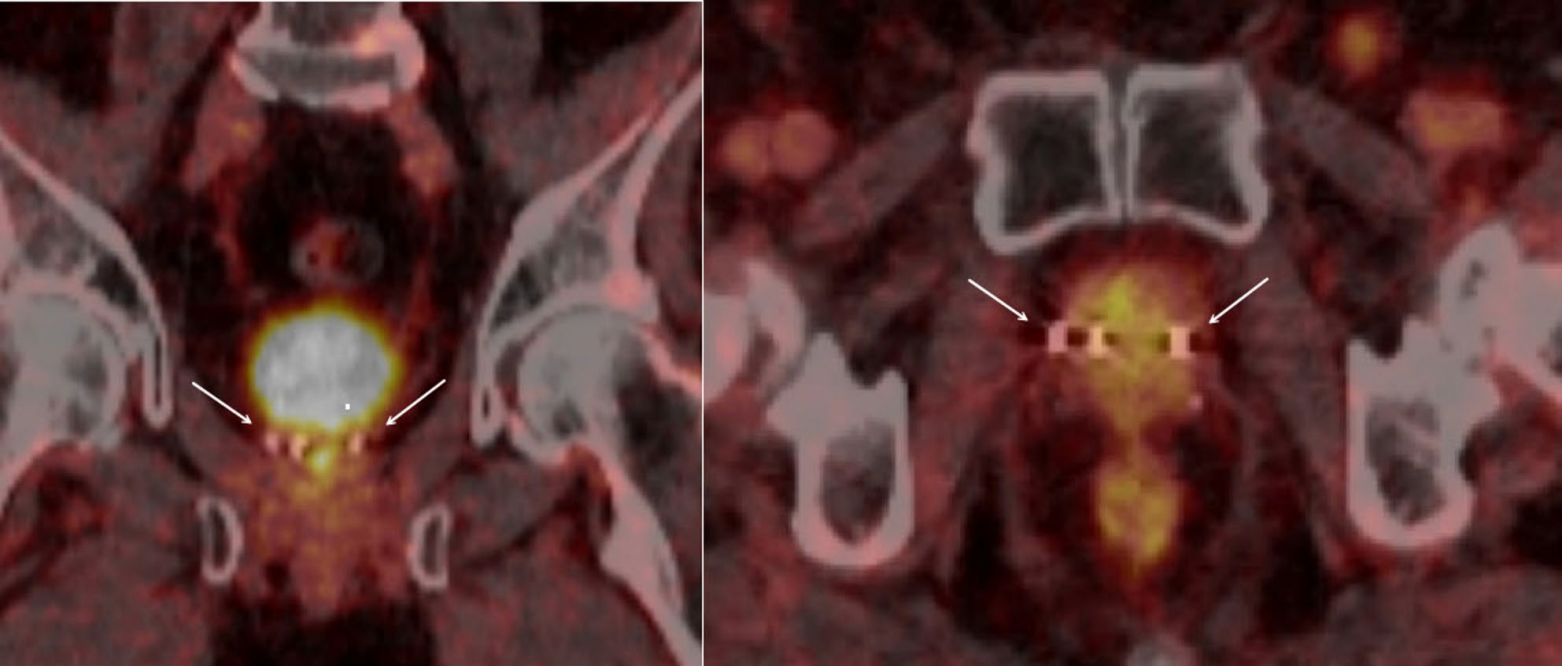
PSMA PET/CT. Left: coronal plane; right: axial plane. The two white arrows indicate the two gold markers

Approximately one year later, in December 2024, a second biochemical recurrence was documented (PSA 1.4 ng/ml). PSMA-PET/CT again demonstrated focal uptake within the prostate without evidence of distant metastases. MRI revealed a size-stable lesion (16 × 11 × 13 mm) in the right anterior mid-gland transitional zone (PI-RADS 5). In both imaging modalities, the two fiducial markers were clearly visible. (Figures 1 and 2) The case was rediscussed at the multidisciplinary tumor board, and rebiopsy to evaluate the extent of recurrence and guide further therapy was recommended.

A transperineal MRI and ultrasound-guided fusion biopsy under local anesthesia and sedation administered by the urologist was performed according to the institutional protocol. Three targeted cores were obtained from the target lesion, along with three perilesional and three contralateral template biopsies.

**Fig. 3:**
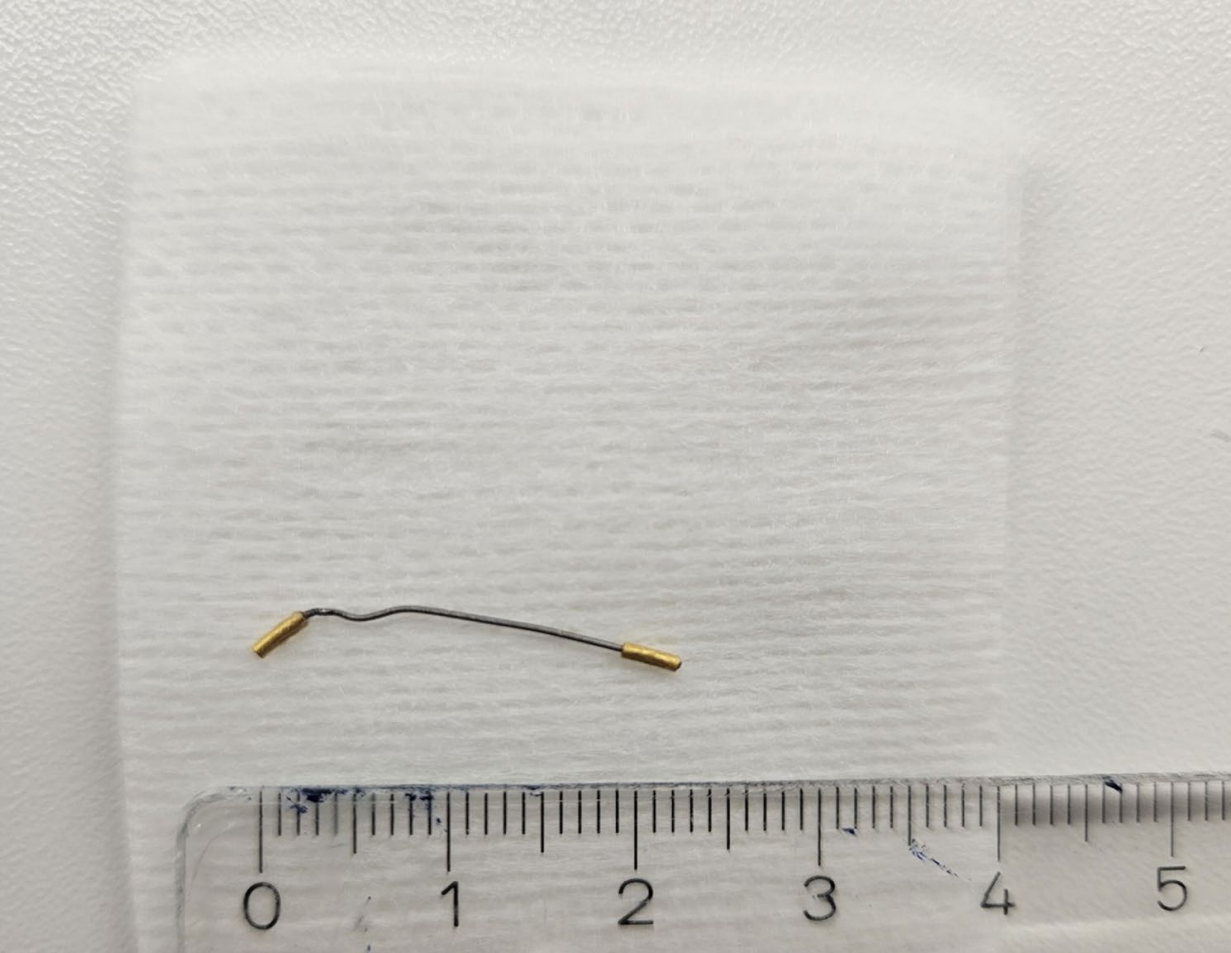
The fiducial gold marker removed

While performing the last biopsy in the left transitional zone at the mid-gland level, increased resistance was encountered during needle advancement. Notably, despite real-time ultrasound guidance, the fiducial marker could not be clearly visualized intraoperatively, likely due to differences in imaging planes relative to the ultrasound probe. Upon needle retraction, a previously implanted gold fiducial marker was found embedded within the biopsy core and extracted percutaneously (Fig. 3). The patient experienced no complications and was monitored postoperatively in accordance with our institutional protocol.

Histopathological analysis of the targeted biopsy revealed prostate adenocarcinoma with a Gleason score of 4 + 5=9 (Grade Group 5). Based on these findings, systemic therapy with androgen deprivation therapy (ADT) in combination with enzalutamide was initiated.

### Transperineal MRI/ultrasound fusion biopsy technique

In our clinical practice, we employ a transperineal approach for prostate biopsies, utilizing the KOELIS Trinity^®^ system. This platform enables real-time 3D mapping of the prostate, integrating preacquired multiparametric MRI data with live ultrasound imaging. Prostate biopsies are performed under local anesthesia, with sedation administered directly by the urologist. Local anesthesia is administered using 40mL 1% mepivacaine to the skin, subcutaneous tissue, and periprostatic area. Sedation is achieved with 7.5 mg of midazolam and inhaled methoxyflurane (Penthrox^®^), provided that there are no contraindications. This combination ensures adequate analgesia and patient relaxation throughout the procedure.

**Fig. 4:**
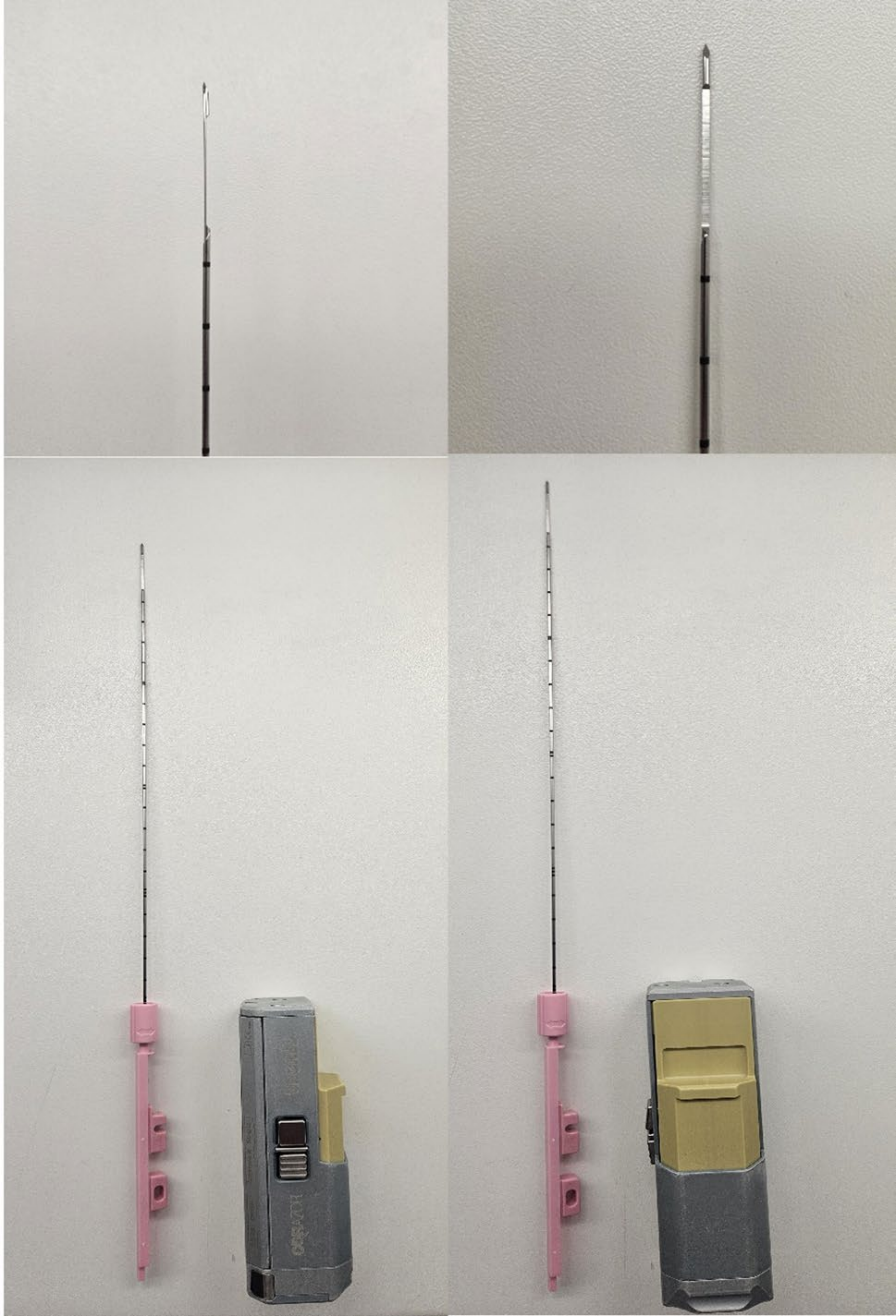
UROMED CORAZOR^®^ biopsy system

Our biopsy strategy is comprehensive: we obtain three targeted cores from each identified lesion and three perilesional samples to assess the surrounding tissue. Additionally, systematic template biopsies are performed on the contralateral side if indicated. Tissue sampling is conducted via the UROMED CORAZOR^®^ biopsy system (Fig. 4), which features high spring tension for powerful puncture and optimized tissue samples. We utilize 240 mm, 18G (1.2 mm) CORAZOR^®^ puncture biopsy cannulas (Fig. 4), which are capable of retrieving tissue cylinders up to 19 mm in length, ensuring high-quality histological specimens.

## Discussion and conclusion

To our knowledge, this case report represents the first documented iatrogenic displacement and transcutaneous removal of a gold fiducial marker during a transperineal prostate biopsy.

Various types of fiducial markers are available for prostate image-guided radiotherapy, differing in material, size, shape, and imaging characteristics. Gold cylindrical seeds remain the most commonly used markers due to their wide availability, radio-opacity on kilovoltage and megavoltage imaging, and overall stability. Typical gold markers range from approximately 3–20 mm in length and 0.75–1.2 mm in diameter. Larger markers may improve automatic image registration but can generate more imaging artefacts, whereas smaller markers reduce artefact at the potential cost of visibility. Alternative materials such as platinum have been proposed to enhance MRI visibility, and non-metallic markers have been developed to reduce CT artefact and potential dosimetric perturbation.

Fiducial marker implantation is generally considered safe, with complications such as infection, bleeding, and marker migration being relatively uncommon [2]. ​Studies have shown that intraprostatic migration of fiducial markers can occur during radiation therapy and is relatively common [6–8]. However, extraprostatic displacement—where markers move beyond the prostate gland—is rare and has not been extensively documented [9].

The dislodgment of a fiducial marker during a transperineal biopsy, as observed in this case, highlights the need to consider biomechanical factors. The use of an 18G biopsy needle, which has a relatively large diameter, may increase the risk of engaging and dislodging a marker, especially if the needle path overlaps with the marker’s location. ​The timing between fiducial marker implantation and subsequent procedures may influence the risk of marker migration. Immediately after insertion, the prostate may experience swelling or bleeding, potentially leading to marker displacement before the tissue has fully healed and secured the markers in place. Interestingly, in our case, the fiducial marker was displaced around 5 years after implantation, suggesting that even long-term stability may not preclude the possibility of migration.

An additional consideration is the potential impact of fiducial markers on MRI interpretation and fusion biopsy accuracy. Gold fiducial markers are known to generate susceptibility artifacts on MRI, which may affect local image quality depending on marker size, composition, and sequence parameters [2]. In our case, although fiducial markers were clearly identifiable, they did not significantly impair lesion detection or PI-RADS assessment, as the index lesion remained well visualized on multiparametric MRI. Awareness of marker location and potential artifact-related distortion is therefore important when planning and performing fusion-guided biopsies.

This case underscores the importance of meticulous planning in patients with existing fiducial markers undergoing further prostate interventions. Preprocedural imaging to ascertain the exact location of markers can aid in avoiding their inadvertent removal. Furthermore, clinicians should be aware of the potential for marker dislodgment. In such scenarios, reimplantation of markers may be necessary. Notably, if the fiducial markers were implanted transrectally, the risk of secondary dislocation during subsequent procedures, such as transperineal biopsies, may be increased, as the two interventions do not follow parallel access routes. To mitigate this risk, careful preprocedural planning to identify marker location is essential, and intraoperatively, operators should remain aware of this possibility and avoid needle trajectories that overlap with known marker positions.

In our patient, the exact brand and specifications of the implanted fiducial markers (including type, length, and diameter) could not be determined despite retrospective attempts to retrieve this information, representing a limitation of this report.

In conclusion, this rare case of transcutaneous fiducial marker removal during a biopsy underscores the importance of considering potential marker displacement even years after implantation. Although clinically uneventful, such occurrences may impact radiotherapy planning and warrant heightened vigilance during subsequent prostate interventions. Further reporting and systematic evaluation of such events are needed to guide clinical practice.

## Data Availability

The data, figures and medical records are included in this case report. Additional details of the remaining data used during the current study are available from the corresponding author upon reasonable request.

## Abbreviations

ADT: Androgen Deprivation Therapy
CT: Computed Tomography
Gy: Gray
IGRT: Image-Guided Radiotherapy
MRI: Magnetic Resonance Imaging
PCa: Prostate Cancer
PET: Positron Emission Tomography
PI-RADS: Prostate Imaging Reporting and Data System
PSA: Prostate-Specific Antigen
PSMA: Prostate-Specific Membrane Antigen
